# Importance of Active Participation in Obesity Management Through Mobile Health Care Programs: Substudy of a Randomized Controlled Trial

**DOI:** 10.2196/mhealth.8719

**Published:** 2018-01-03

**Authors:** Bumjo Oh, Ga-Hye Yi, Min Kyu Han, Jong Seung Kim, Chang Hee Lee, Belong Cho, Hee Cheol Kang

**Affiliations:** ^1^ Department of Family Medicine Seoul Metropolitan Government - Seoul National University Boramae Medical Center Seoul National University College of Medicine Seoul Republic Of Korea; ^2^ SK International Medical Center Fenghuang Hospital Wuxi China; ^3^ Future IT R&D Laboratory LG Electronics Seoul Republic Of Korea; ^4^ Department of Family Medicine Seoul National University Hospital Seoul National University College of Medicine Seoul Republic Of Korea; ^5^ Department of Family Medicine Severance Hospital Yonsei University College of Medicine Seoul Republic Of Korea

**Keywords:** physical activity, mobile health, metabolic syndrome, self-report, adherence, concordance

## Abstract

**Background:**

Due to the prevalence of the westernized dietary pattern and lack of physical activity, the numbers of overweight or obese individuals are increasing, resulting in a growing health burden because of various related diseases. A lifestyle modification approach has additional advantages compared with pharmacological therapies or bariatric surgery. In our randomized controlled trial conducted in 2015, we successfully used a ubiquitous health care (SmartCare) service for patients with metabolic syndrome to achieve a significant weight loss effect. Various useful apps have been developed for the SmartCare Service, which involves using a mobile phone to manage chronic diseases, minimizing time and space restrictions. Many studies have demonstrated weight loss effects using a SmartCare service, but limited data are available regarding the effect of active participation in relation to weight loss.

**Objective:**

We aimed to assess the weight loss effect achieved after using the SmartCare service in terms of adherence and participation. We divided the intervention group of the previous study according to participation level, and analyzed whether there was a significant difference in the outcome.

**Methods:**

We classified participants into 3 groups according to their adherence. Within the intervention group using the SmartCare service, the active group comprised those transmitting anthropometric measurement data using a mobile phone 3 or more times per week or who had a health consultation 5 or more times during a 24-week period. The passive group comprised those who did not adhere to these levels of engagement. The control group comprised those who did not use the SmartCare service. We compared changes in body weight, body mass index (BMI), body fat percentage, waist circumference, and lipid profile among the 3 groups.

**Results:**

We identified 422 participants and analyzed 405, excluding 17 who were missing necessary data for analysis. The active group consisted of 116 participants, compared with 80 in the passive group and 209 in the control group (without SmartCare service). There was a statistically significant difference in improvements to body weight, BMI, body fat percentage, and waist circumference among active participants compared with less active participants and the control group (*P*<.05). However, the lipid profile changes did not differ significantly between groups.

**Conclusions:**

The level of participation may be related to improved weight-related outcomes, which may improve health outcomes. To maximize the effectiveness of the SmartCare service, encouraging active participation is important.

**Trial Registration:**

Clinicaltrials.gov NCT01344811; https://clinicaltrials.gov/ct2/show/NCT01344811 (Archived by WebCite at http://www.webcitation.org/6alT2MmIB)

## Introduction

Being overweight or obese increases the risk of cardiovascular disease, diabetes, cancer, and musculoskeletal disorders, resulting in approximately 3 million deaths worldwide each year [1]. Westernized dietary patterns and a lack of physical activity are considered to be the primary causes. According to the Korea National Health and Nutrition Examination Surveys, the prevalence of obesity in Korean adults was 32.0% in 2011, which was much higher than the 26.9% recorded in 1998 [2]. Therefore, it is important to manage and treat obesity, and it is essential to improve eating and exercise habits before considering medication or surgical treatment options [3]. However, because lifestyle modifications need continuous monitoring and feedback in everyday life, patients can find these modifications difficult and burdensome [4]. An important issue concerns how best to overcome the difficulties in adhering to lifestyle modifications.

Koreans have the highest level of smartphone ownership in the world, and smartphone penetration rate reached 79.5% in 2012 [5]. Recently, the use of a ubiquitous health care (SmartCare) service, equipped with advanced technology for the health care of chronically ill patients with hypertension and diabetes, has been rapidly growing [6]. Numerous studies have revealed that a mobile phone can be used as a strategic tool for weight management in obese patients [7-9]. Unlike desktop computers, a mobile phone provides mobility to users, and also includes sensors such as global positioning systems and acceleration sensors, as well as being applicable to various mobile health care services. It is also possible to access the Internet easily without time and space restrictions, thus enabling real-time one-on-one consultation and communication with health care experts.

Previously, we conducted a randomized controlled trial to evaluate the effect of a SmartCare service on weight management in obese patients with metabolic syndrome (NCT01344811) [10]. Among adults over 20 years of age who visited Seoul National University Hospital or the Severance Hospital, both in Seoul, South Korea, obese patients with metabolic syndrome were identified for possible inclusion in this study. The prevalence of metabolic syndrome in Korean adults continues to increase, which increases cardiovascular morbidity and mortality and all-cause mortality [11-13].

That study was, to our knowledge, the first government-funded project in Korea undertaken to estimate the usefulness of a SmartCare service in managing chronic disease. It concluded that weight control using the SmartCare service was significantly better than for a control group using conventional methods. However, the effect of adherence to the SmartCare service has not been analyzed among participants.

Recent studies related to mobile health care services have focused primarily on comparing information and communication technology intervention efficacy between users and nonusers, so there is little research on whether adherence and weight loss outcomes are positively correlated. The purpose of this study was to clarify the relationship between adherence and weight loss outcomes through further analysis of our previous study.

Prior to the study, we hypothesized that a higher level of participation would produce a better outcome, and we attempted to set criteria for intensive participation. As family medical practitioners involved in patient education, we aimed not only to recommend the SmartCare service, but also to provide effective participation criteria.

## Methods

### Recruitment

We advertised our study using institutional review board (IRB)-approved banners, posters, and leaflets placed in the hospitals’ lobbies.

According to the Adult Treatment Panel III criteria, using waist circumference cutoff modifications for Asian populations as suggested in the Asia-Pacific guidelines, we defined metabolic syndrome as having 3 or more of the following factors [14]: (1) central obesity: waist circumference 90 cm or more in men and 80 cm or more in women; (2) hypertriglyceridemia: triglyceride 8.325 mmol/L or more; (3) high-density lipoprotein (HDL) cholesterol less than 2.22 mmol/L in men and less than 2.775 mmol/L in women; (4) hypertension: blood pressure 130/85 mmHg or more, or taking antihypertensive medication; and (5) hyperglycemia: fasting glucose 5.55 mmol/L or more, or taking antidiabetic medication.

The World Health Organization Western Pacific Region recommends defining obesity in Asian populations as those individuals with a body mass index (BMI) 25 kg/m^2^ or greater [15]. The Korean Society for the Study of Obesity also investigated BMI cutoff figures for obesity-related disease and adopted the World Health Organization’s definition. Nowadays, Korean government organizations officially use this definition when defining and implementing health policies regarding obesity in Korea [16].

However, we excluded from this study individuals taking thyroid hormone or antiobesity medication, which can affect weight, along with patients with insulin-dependent diabetes with liver functional abnormality (with a liver somatic index over 3 times higher than the normal maximum level) or with renal functional impairment (with a creatinine level over one and a half times higher than the normal maximum level), pregnant women, and inpatients.

We assigned eligible participants to 2 groups, with equal probability, according to a randomization code. The randomization code was prepared using a block randomization method, stratified by a statistician in a clinical trial center (C&R Research, Seoul, South Korea). As this study was an open-label trial, blinding was not undertaken.

The IRB of Seoul National University Hospital approved this study (IRB number: h-1009-095-333).

### Intervention Using a SmartCare Service

We randomly allocated participants into 2 groups. The intervention group was provided with a mobile phone equipped with a SmartCare app, a body composition meter (InBody IH-U070B, InBody Co Ltd, Seoul, South Korea) with Bluetooth function, and a pedometer (MP-100, Yamasa Co, Ltd, Okayama, Japan).

Each participant measured his or her own body weight and body composition (skeletal muscle mass, fat mass) using the provided body composition monitor at the same hour every day, if possible (or at least 3 times a week), after waking up and before breakfast. After measuring the relevant values with the body composition monitor, the participants placed the transmission terminal (Bluetooth) of the remote monitoring device near the transmission terminal of the mobile phone to transmit the measurement data to the central server in the SmartCare center via mobile phone (wireless network). Each participant carried a pedometer on the waist from the time they woke until the time they went to bed. Participants checked their activity level, indicated as the number of steps taken, at the same time every day, if possible, and entered this into the mobile phone (data entry before going to bed was recommended). The entered data were then automatically transmitted to the central server in the SmartCare Center. Physicians or health care personnel at the SmartCare Center could retrieve hospital admission information, treatment records, diagnosed diseases identification, diagnostic examinations and functional test results, and prescription information of the test participants through connecting to the hospital information system, with participant consent. The central server in the SmartCare Center transmitted the feedback, based on the measured body weight and body composition, to the mobile phones of the participants, according to the algorithm of the clinical decision support system. The participants were able to immediately check the interpretations and recommendations, based on their measured values, through their mobile phones.

**Figure 1:**
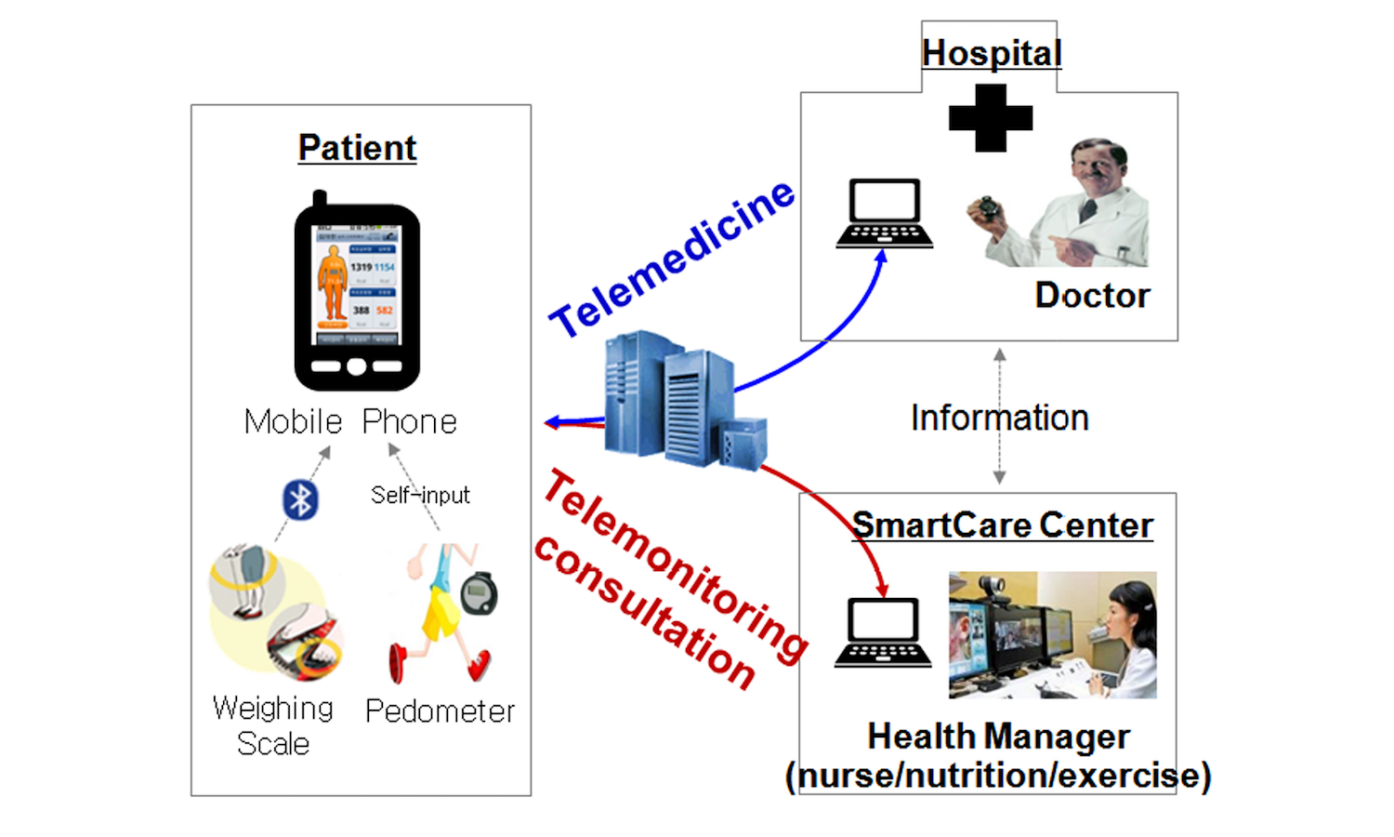
Model of SmartCare service.

Trained consultants (nurses, exercise specialists, and clinical dietitians) at the SmartCare Center undertook various health consultations through the patients’ telephone inquiries concerning disease management, health education, recommended exercise, medication, and appropriate nutrition. Monthly and weekly health reports, based on the individual patient’s measured values and daily living records, were sent directly to the patients through the SmartCare system. Additionally, in instances where medical treatment was received at Seoul National University Hospital or the Severance Hospital, the physician providing the medical care had access to the information (Figure 1).

### Comparison Group

We provided the comparison group only with anthropometric instruments and pedometers.

We provided body weight scales and pedometers to the participants assigned to the control group. Body weight journals were distributed to the participants, and each participant self-measured and recorded his or her daily weight and waist size (a minimum of 3 times per week) at the same time (before breakfast). They also wore a pedometer during daily activities, which started from the time they woke in the morning until bedtime. They were instructed to check and record their daily walking distances on the record sheet just before going to bed. Additionally, the participants in the control group had the same hospital visit schedule as that of the intervention group at 12- and 24-week intervals, and received anthropometry, consultations with physicians, and information about their nutrition and exercise.

As with the intervention group, individual health care counseling services were provided, but only at the hospital.

As an incentive for registering as study participants for both the intervention and comparison groups, the Korean Ministry of Commerce, Industry and Energy national project budget covered all expenses for medical treatment, medication, transportation, and communication (mobile phone provision and use).

### Study Design

**Figure 2:**
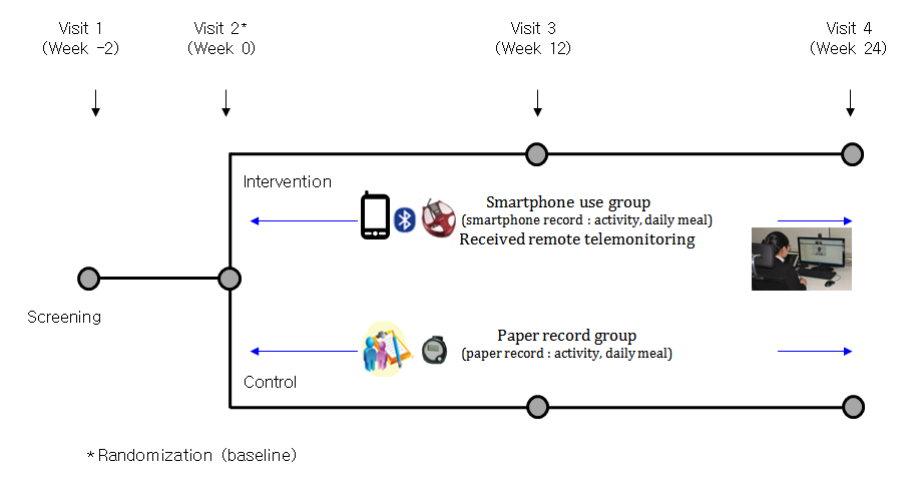
Flowchart of the intervention.

We requested the participants to visit the hospitals 4 times during the 24-week period. Except when screening was performed, body weight, body composition, and blood pressure measurements were recorded. A hematology test was performed, and changes in their life habits (eg, diet intake and physical activity) were recorded 3 times during the test period: once on the date of random allocation, once in week 12, and once in week 24 (Figure 2).

**Figure 3:**
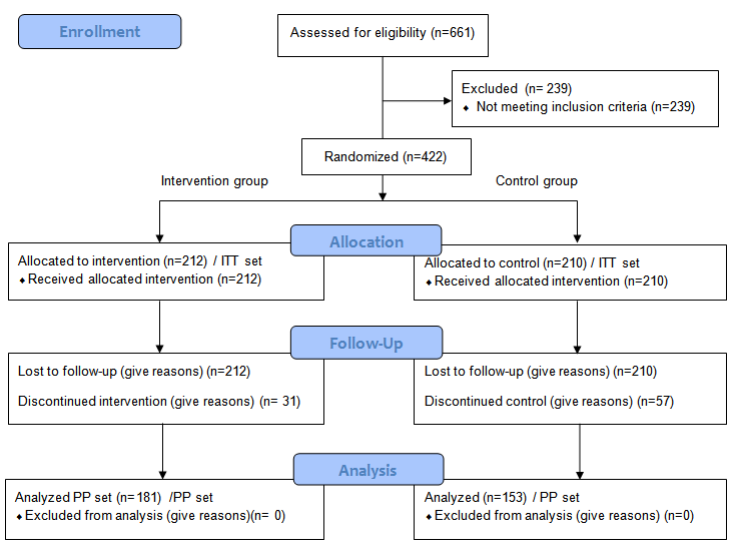
Selection of the study participants. ITT: intention-to-treat; PP: per protocol.

Analysis consisted of 2 group sets: intention-to-treat (ITT) and per protocol. The ITT set included all participants who were enrolled in this clinical trial and were randomly allocated. When analyzing efficacy, we included participants in the treatment group to which they had been randomly allocated, regardless of the actual treatment they received. Among the participants in the ITT set, we included those who completed this clinical trial without a material breach of the protocol in the per protocol set (Figure 3).

### Additional Analysis According to Medical Adherence

We classified the intervention group into an active participation group and a passive participation group, according to adherence. The definition of adherence was divided into 2 parts as follows: (1) measurement of anthropometric data and input of pedometer data directly into the mobile phone 3 or more times a week, and (2) taking part in a health consultation 5 or more times during a 24-week period.

### Measurement Variables

At the time of screening, demographic information (age, sex, smoking, alcohol consumption, and other relevant information), medical history, and medication history of the participants were investigated and recorded. Additionally, an electrocardiogram was recorded at the screening visit after the participant had been resting for at least 5 minutes. When test results were clinically significant, the investigator determined whether to enroll the participant in the study.

Laboratory tests were conducted at screening, baseline, week 12, and week 24 for alanine aminotransferase, aspartate aminotransferase, creatinine, lipid profile (total cholesterol, HDL cholesterol, and triglyceride), fasting glucose, and hemoglobin A_1c_. However, alanine aminotransferase, aspartate aminotransferase, and creatinine tests were performed only at screening to determine trial eligibility. Lipid profiles and blood glucose tests were performed after the participant had been fasting.

We evaluated weight change, the primary outcome, using body fat percentage at baseline and at 12 and 24 weeks, as measured by nurses using portable bioelectrical impedance analysis (InBody U20, InBody Co Ltd).

We assessed and categorized the level of physical activity using the International Physical Activity Questionnaire at baseline, 12 weeks, and 24 weeks. The amount of physical activity each week was calculated with a continuous variable, namely the metabolic equivalent task (MET), as follows: total METs min/week = [walking METs × min × days] + [moderate METs × min × days] + [vigorous METs × min × days]) [17].

For measuring caloric intake variables, daily meal record cards (3-day recall dietary assessments) were distributed to the participants during their initial visit, and the participants were instructed to write their own 3-day meal record just before the baseline visit, the next visit after 12 weeks, and the final visit at 24 weeks. The self-completed daily meal records were collected from the participants during their final visit, and dietitians calculated caloric intake using the nutrient evaluation program CAN-Pro 3.0 (The Korean Nutrition Society) [18].

### Statistical Analysis

The effectiveness analysis comprised the ITT population, which included all randomly allocated participants. We imputed missing data using the last observation carried forward method.

We calculated descriptive statistics (number of observed participants, mean [standard deviation (SD)], and median [range]) on body weight measured at baseline and at the end point (24 weeks), and the changes in measured values at week 24 compared with baseline for each group. To identify the difference between the groups with respect to body weight changes at week 24 compared with baseline, we used analysis of covariance (ANCOVA), with the clinical trial institution that recruited participants and body weight at baseline as covariates.

For continuous data (changes in BMI, body fat percentage, waist measurement, lipid profile, blood pressure, number of metabolic syndrome elements, diet intake in kilocalories, physical activity, number of steps taken, and weight to measure physical activity), we determined descriptive statistics (number of observed participants, mean [SD], and median range]) per group. To identify the difference between the groups in the changes at week 24 compared with baseline, we performed ANCOVA or rank transformation ANCOVA. When conducting ANCOVA, we set the clinical trial institution that recruited participants and the baseline values of relevant parameters as covariates.

All analyses were conducted using SAS version 9.3 (SAS Institute). All statistical tests were performed at a 2-sided significance level of .05.

## Results

### Baseline Characteristics

We identified 422 patients able to use mobile phones, and who were willing to participate in the study and sign informed consent forms, for recruitment. All participants received the SmartCare service, and we excluded from the final analysis 17 participants who did not enter any data for 24 weeks. Following the definition of adherence already described, we used 2 criteria for dividing the active and passive participation groups. First, we analyzed the demographic characteristics according to the number of weekly mean anthropometric measurements. Second, we analyzed the data according to total counseling frequency, age, sex, BMI, alcohol consumption, education level, and smoking status did.

### Efficacy Evaluation Summary

#### Changes in Anthropometric Data During the 24-Week Period

**Figure 4:**
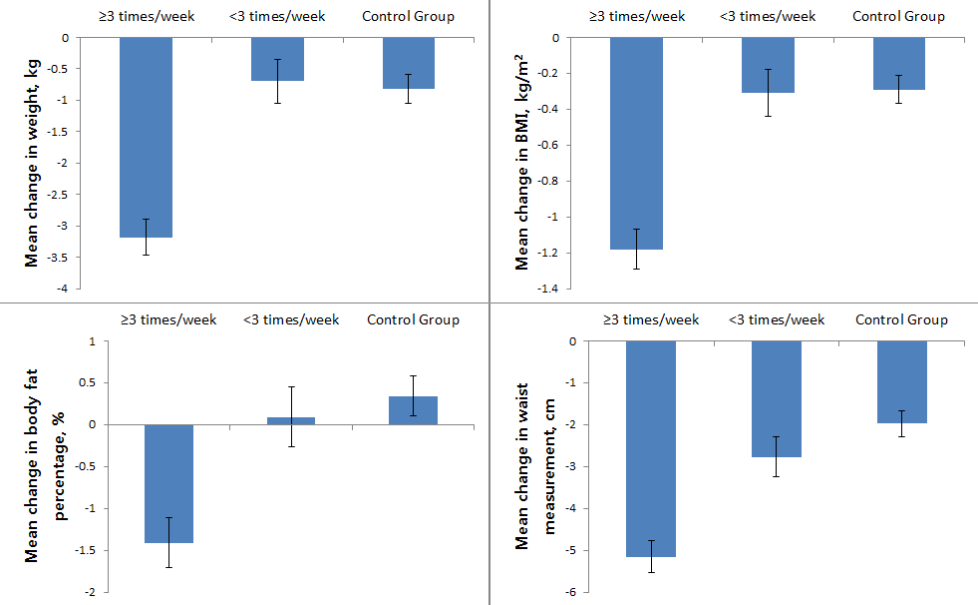
Changes in anthropometric data during the 24-week period analyzed according to weekly mean number of anthropometric measurements. Error bars indicate standard deviation. BMI: body mass index.

First, we examined differences in characteristics according to the weekly mean number of anthropometric measurements (Figure 4).

Following classification according to self-reporting frequency, there were 116 active participants and 80 less active participants (Table 1). The average weight loss effect during the whole period was greatest in the active participation group, lower in the control group, and lowest in the less active participation group. There was no statistically significant difference between the control group and the less active participation group (Table 1). The mean change of body weight was –3.18 (SD 0.29) kg in the active group, –0.70 (SD 0.35) kg in the less active group, and –0.82 (SD 0.23) kg in the control group. BMI, body fat percentage, and waist circumference were most enhanced for those in the active participation group, less enhanced in the less active participation group, and least enhanced in the control group. In particular, there was no difference in body weight and BMI at baseline between the active participant group and the control group, but there was a statistically significant difference in body weight, BMI, body fat percentage, and waist circumference at the end of 24 weeks (Table 1).

**Table 1:** Demographic and other pretreatment characteristics (intention-to-treat set) by the weekly mean number of anthropometric measurements (n=405).

Demographic and other pretreatment characteristics			Intervention group		Control group (C)	Between-group *P* value		
			≥3 times a week (A)	<3 times a week (B)		A-B	A-C	B-C
**Age (years)**								
	Total, n (%)		116 (28.6)	80 (20.0)	209 (51.6)			
	Mean (SD^a^)		50.45 (12.24)	42.11 (12.33)	50.36 (14.27)	<.001^b^	.96^b^	<.001^b^
	Median		52.50	41.50	52.00			
	Range		23.00-71.00	20.00-72.00	21.00-82.00			
	**Range, n (%)**							
		20-29	5 (4.3)	14 (17.5)	18 (8.6)	<.001^c^	.55^c^	.001^c^
		30-39	21 (18.1)	23 (28.8)	40 (19.1)			
		40-49	21 (18.1)	20 (25.0)	33 (15.8)			
		50-59	40 (34.5)	15 (18.8)	61 (29.2)			
		≥60	29 (25.0)	8 (10.0)	57 (27.3)			
**Sex, n (%)**								
	Total		116 (28.6)	80 (20.0)	209 (51.6)			
	Male		57 (49.1)	44 (55.0)	101 (48.3)	.42^c^	.89^c^	.31^c^
	Female		59 (50.9)	36 (45.0)	108 (51.7)			
**Body mass index (kg/m^2^)**								
	Total, n (%)		116 (28.6)	80 (20.0)	209 (51.6)			
	Mean (SD)		28.77 (3.09)	29.72 (3.11)	29.40 (3.39)	.03^b^	.1^b^	.45^b^
	Median		28.00	29.20	28.90			
	Range		24.90-41.00	25.00-41.40	24.90-41.80			
**Body weight (kg)**								
	Total, n (%)		116 (28.6)	80 (20.0)	209 (51.6)			
	Mean (SD)		77.48 (12.30)	83.86 (14.51)	79.74 (15.28)	.001^b^	.15^b^	.04^b^
	Median		76.60	83.20	76.90			
	Range		55.20-128.50	58.60-135.40	54.00-141.10			
**Height (cm)**								
	Total, n (%)		116 (28.6)	80 (20.0)	209 (51.6)			
	Mean (SD)		163.82 (8.73)	167.49 (9.29)	164.12 (10.61)	.005^b^	.78^b^	.01^b^
	Median		165.00	169.20	163.00			
	Range		146.00-182.00	144.50-188.90	142.80-189.00			
**Smoking status, n (%)**								
	Total		115 (28.4)	80 (20.0)	203 (51.6)			
	Nonsmoker		72 (62.6)	47 (58.8)	129 (63.6)	.02^c^	.02^c^	.65^c^
	Former smoker		34 (29.6)	16 (20.0)	40 (19.7)			
	Smoker		9 (7.8)	17 (21.3)	34 (16.8)			
**Drinking status, n (%)**								
	Total		115 (28.4)	80 (20.0)	206 (51.6)			
	Nondrinker		51 (44.4)	20 (25.0)	84 (40.8)	.01^c^	.68^c^	.03^c^
	Former drinker		10 (8.7)	5 (6.3)	15 (7.3)			
	Drinker		54 (47.0)	55 (68.8)	107 (51.9)			
**Completed education level, n (%)**								
	Total		116 (28.6)	80 (20.0)	209 (51.6)			
	Elementary school		4 (3.5)	0	21 (10.2)	.11^d^	.15^c^	.001^d^
	Middle school		6 (5.2)	3 (3.85)	15 (7.2)			
	High school		39 (33.6)	19 (23.8)	65 (31.1)			
	University		67 (57.8)	58 (72.5)	108 (51.7)			

^a^SD: standard deviation

^b^2-sample *t* test.

^c^Pearson chi-square test.

^d^Fisher exact test.

**Figure 5:**
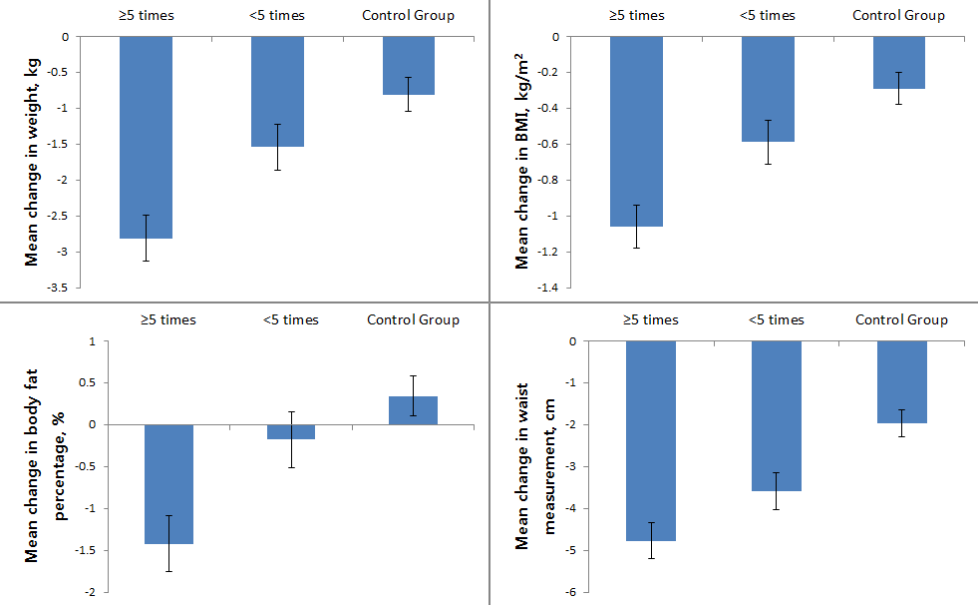
Changes in anthropometric data during the 24-week period analyzed according to total number of health consultations. Error bars indicate standard deviation. BMI: body mass index.

Second, we examined differences in characteristics according to the total number of health consultations (Figure 5).

Following classification according to the total number of health consultations, there were 98 active participants and 98 less active participants (Table 2). Weight, BMI, body fat percentage, and waist circumference were highest in the active participation group, lower in the less active participation group, and lowest in the control group. The mean change of body weight was –2.81 (SD 0.32 kg in the active group, –1.54 (SD 0.32) kg in the less active group, and –0.81 (SD 0.24) kg in the control group. In addition, improvements in body weight, BMI, body fat percentage, and waist circumference differed significantly between the active and the less active participation groups. Even where participants received the same SmartCare service, it was clear that the outcome depended on the participation level (Table 2).

#### Changes in Biomarker (Lipid Panel) During the 24-Week Period

When we analyzed biomarkers according to the weekly mean number of anthropometric measurements and the total number of health consultations, we found no statistically significant differences between groups (data not shown).

**Table 2:** Demographic and other pretreatment characteristics (intention-to-treat set) by total number of health consultations (n=405).

Demographic and other pretreatment characteristics			Intervention group		Control group (C)	Between-group *P* value		
			≥5 times in total period (A)	<5 times in total period (B)		A-B	A-C	B-C
**Age (years)**								
	Total, n (%)		98 (24.2)	98 (24.2)	209 (51.6)			
	Mean (SD^a^)		49.45 (12.29)	44.64 (13.14)	50.36 (14.27)	.009^b^	.59^b^	.001^b^
	Median		50.50	44.00	52.00			
	Range		23.00-72.00	20.00-71.00	21.00-82.00			
	**Range, n (%)**							
		20-29	6 (6.1)	13 (13.3)	18 (8.6)	.17^c^	.67^c^	.07^c^
		30-39	19 (19.4)	25 (25.5)	40 (19.1)			
		40-49	19 (19.4)	22 (22.5)	33 (15.8)			
		50-59	33 (33.7)	22 (22.5)	61 (29.2)			
		≥60	21 (21.4)	16 (16.3)	57 (27.3)			
**Sex, n (%)**								
	Total		98 (24.2)	98 (24.2)	209 (51.6)			
	Male		53 (54.1)	48 (49.0)	101 (48.3)	.48^c^	.35^c^	.91^c^
	Female		45 (45.9)	50 (51.0)	108 (51.7)			
**Body mass index (kg/m^2^)**								
	Total, n (%)		98 (24.2)	98 (24.2)	209 (51.6)			
	Mean (SD)		28.82 (3.30)	29.50 (2.93)	29.40 (3.39)	.13^b^	.16^b^	.8^b^
	Median		28.00	29.00	28.90			
	Range		25.00-41.40	24.90-37.90	24.90-41.80			
**Body weight (kg)**								
	Total, n (%)		98 (24.2)	98 (24.2)	209 (51.6)			
	Mean (SD)		79.00 (13.83)	81.17 (13.30)	79.74 (15.28)	.26^b^	.68^b^	.46^b^
	Median		77.25	80.95	76.90			
	Range		55.20-128.50	55.50-135.40	54.00-141.10			
**Height (cm)**								
	Total, n (%)		98 (24.2)	98 (24.2)	209 (51.6)			
	Mean (SD)		165.17 (8.79)	165.47 (9.48)	164.12 (10.61)	.82^b^	.36^b^	.28^b^
	Median		167.00	165.35	163.00			
	Range		144.50-181.00	148.00-188.90	142.80-189.00			
**Smoking status, n (%)**								
	Total		98 (24.2)	97 (24.2)	203 (51.6)			
	Nonsmoker		57 (58.2)	62 (63.9)	129 (63.6)	.01^c^	.01^c^	.87^c^
	Former smoker		33 (33.7)	17 (17.5)	40 (19.7)			
	Smoker		8 (8.2)	18 (18.6)	34 (16.8)			
**Drinking status, n (%)**								
	Total		98 (24.2)	97 (24.2)	206 (51.6)			
	Nondrinker		33 (33.7)	38 (39.2)	84 (40.8)	.36^c^	.41^c^	.72^c^
	Former drinker		10 (10.2)	5 (5.2)	15 (7.3)			
	Drinker		55 (56.1)	54 (55.7)	107 (51.9)			
**Completed education level, n (%)**								
	Total		98 (24.2)	98 (24.2)	209 (51.6)			
	Elementary school		1 (1.0)	3 (3.1)	21 (10.1)	.37^d^	.02^c^	.04^d^
	Middle school		4 (4.1)	5 (5.1)	15 (7.2)			
	High school		34 (34.7)	24 (24.5)	65 (31.1)			
	University		59 (60.2)	66 (67.4)	108 (51.7)			

^a^SD: standard deviation

^b^2-sample *t* test.

^c^Pearson chi-square test.

^d^Fisher exact test.

## Discussion

### Principal Findings

One previous study found that self-monitoring of body weight in the workplace had a preventive effect on weight gain [19]. That study concluded that just increasing the self-measurement frequency of body weight had a preventive effect, that is, weight management improved through measurement adherence. For this reason, we wanted to identify the cutoff value of adherence, given that people with better adherence during the 24-week study period had a better outcome. As a measure of participation, we identified the weekly mean number of anthropometric measurements and the total number of health consultations. As we had hypothesized, the higher the participation, the greater the improvement in anthropometric measurements.

The high frequency of physical measurement and consultation frequency indicates that there was a strong willingness among participants to manage their obesity.

Health care information technology (IT), also referred to as medical IT, encompasses the process of storing, analyzing, and delivering information, data, and knowledge from all activities related to health care, using information processing technology and networks. Demand is rapidly increasing in line with developments in areas of medical care, such as population aging, early diagnosis and treatment of diseases, and preventive medicine. The medical IT convergence technologies that are being actively developed in Korea include home care, using a blood pressure monitor and blood glucose meter; home health care, using a mobile phone to measure and transmit biometric information to a service center; and wearable health care, involving a wearable biosignal measurement system [20].

Many studies have already demonstrated the utility of managing chronic diseases and obesity through health care programs that use IT [21-26]. However, because programs are implemented outside the hospital, continuous monitoring is needed to encourage active participation, and it is important to inform patients that the higher the participation, the better the health improvement.

### Comparison With Prior Research

Many studies have shown that close adherence to medical treatment, or to lifestyle modification, results in a favorable outcome [27-29]. Mobile phone health care is a new strategic tool for chronic disease management, and numerous studies have demonstrated the effectiveness of mobile phone health care. However, our hypothesis was that active participation would maximize the effects of mobile phone health care, about which there has been little previous research. In our study, we defined adherence as measuring anthropometric data 3 or more times a week, or taking part in voluntary health consultations at least 5 times in 24 weeks. However, the definition of adherence can vary [30-32]. Further studies should be conducted to demonstrate the relevance of adherence and the effectiveness of mobile health care.

### Limitations and Strengths

Concerning the lipid profile, although participation in the SmartCare service was high, the improvement effect was not significant. Previous studies have attempted to improve patients’ lipid profile through lifestyle modification, and these studies have usually involved following the changing pattern of biomarkers from 3 months to 5 years [33,34]. It is possible that the 24-week period set in this study was not sufficient to show changes in lipid profiles. Another plausible reason for the reduced improvement in lipid profiles is that the intervention to improve dietary habits was not appropriately targeted. A more active intervention in respect of eating habits could also improve biomarkers, including lipid profiles.

Although there was no statistically significant difference, all total cholesterol, low-density lipoprotein cholesterol, HDL cholesterol, and triglyceride levels tended to decrease. We found that changes in HDL cholesterol and triglyceride levels, both of which are key factors of metabolic syndrome, were close to a *P* value of .05, although not significant, demonstrating that the SmartCare service may be able to help patients with metabolic syndrome make effective lifestyle modifications.

### Conclusions

Through initial and subsequent studies, we conclude that using the SmartCare service is an effective way to control the weight of obese patients with metabolic syndrome.

In addition, the improvement effect depended on adherence, defined as entering anthropometric information 3 or more times a week or participating in voluntary health consultations at least 5 times in 24 weeks.

## Abbreviations

ANCOVA: analysis of covariance
BMI: body mass index
HDL: high-density lipoprotein
IRB: institutional review board
IT: information technology
ITT: intention-to-treat
MET: metabolic equivalent task
SD: standard deviation
